# Supplementary material for: Gradient polaritonic surface with space-variant switchable light-matter interactions in 2D moiré superlattices
Source: Sci Adv. 2024 Dec 13;10(50):eadq7445. doi: 10.1126/sciadv.adq7445 (PMC11641106; doi:10.1126/sciadv.adq7445)
Supplement: Supplementary file 1 — Supplementary Text Figs. S1 to S12 References [file sciadv.adq7445_sm.pdf]

Science Advances
AAAS

Supplementary Materials for

Gradient polaritonic surface with space-variant switchable light-matter interactions in 2D moiré superlattices

Zhen-Bing Dai *et al.*

Corresponding author: Yue Zhao, zhaoy@sustech.edu.cn; Zhiqiang Li, zhiqiangli@scu.edu.cn

Sci. Adv. **10**, eadq7445 (2024)
DOI: 10.1126/sciadv.adq7445

This PDF file includes:

Supplementary Text
Figs. S1 to S12
References

Supplementary Text

Contents

S1 Device fabrication
S2 Methods for obtaining the experimental $s(x,\varphi)$ profiles
S3 Band structure and local optical conductivity calculations for solitons in bilayer graphene
S4 Simulations of line-profiles, polariton field distributions and near-field images
S5 Extended results demonstrating polariton manipulation by soliton angle profiles
S6 Extended data from a typical mTBG/hBN/SiO_2/Si sample

S1 Device fabrication

For some devices, we used a thin mica substrate with graphite and hBN stacks for further pickup of twisted bilayer graphene, so that the final device has an effective backgate as well as a fresh clean surface. The detailed fabrication process is as following. We first use PC/PDMS stamp to pick up FLG and hBN pieces to form a stack of FLG/hBN (~20 nm)/FLG. The stack was released on top of a centimeter-sized mica substrate at 180 °C, as shown in fig. S1A. We then deposited metal leads, two for TBG and two for the bottom gate, to establish electrical contact from the FLG pieces using e-beam lithography, as shown in fig. S1B. The surfaces of hBN and FLG were cleaned using AFM probe. Next, we flipped the mica and mounted it on PDMS/glass, as illustrated in fig. S1C. Due to its transparency and flexibility, this mica/PDMS/glass can be used as a new stamp for the subsequent pick-ups. Using a "tear and stack" method (43), we picked up a TBG on the stamp from a monolayer graphene crystal exfoliated on SiO_2/Si substrate. The final TBG/FLG/hBN/FLG van der Waals stack on the mica substrate are shown in fig. S1F.

S2 Methods for obtaining the experimental $s(x,\varphi)$ profiles

S2.1 Soliton network of the sample (device B) in fig. S3

As shown in fig. S2, with decreasing gate voltage, the polariton interference feature in the near-field profile $s(x)$ across single solitons with φ~90° evolves from a double-peak to a dip feature (V_g=0 V) centered at the soliton, corresponding to dark lines in the nano-IR image. Fig. S3 shows the near-field amplitude images of a representative sample (device B) with strong lattice distortion at various excitation frequencies and $V_g=0$ (low doping). Region 1 of fig. S3A acquired at ω=1540 cm^{-1} is dominated by dark lines (dip features in $s(x)$ profiles) in the vertical direction only, while region 2 shows dark lines in two directions, both of which are in stark contrast to the typical triangular network consisting of three 1D arrays of dark lines observed in region 3. These dark lines are soliton-induced polariton interference patterns induced by single solitons. The intersections of the dark lines are regions with AA stacking, which are observed as dark spots in the image. As the laser frequency changes from 1540 to 1500 cm^{-1} (fig. S3C), the triangular network of dark lines in region 3 evolves into a hexagonal pattern of dark spots observed at AA sites of mTBG, whereas region 1 (2) displays 1D striped features (1D striped features superposed with dark spots at AA sites). Note that Fig. 4B of the main text is part of fig. S3C. The features at 1500 cm^{-1} arise from the constructive and destructive interference of propagating hBN phonon polaritons triggered by the soliton network (21), since the polariton wavelength is larger than the

moiré period at this frequency. Specifically, the striped feature and hexagonal pattern in fig. S3C arise from polariton interference under the influence of one and three array(s) of nearly shear solitons, respectively. The observed near-field patterns can be analyzed by a superposition model (21) (see Supplementary Information section 4 for details). The simulated near-field images (figs. S3E and F) accurately capture the key features of the experimental images in fig. S3C.

The soliton network of the entire region in fig. S3 can be determined by tracing the soliton-induced features (dark lines) throughout this area as follows. Firstly, the three 1D soliton arrays of the network can be identified from the nearly perfect sixfold triangular network in region 3 of fig. S3A, which provides the three estimated $\Delta\boldsymbol{u}$ directions for the soliton network. From region 3 to the left side of the images, all dark lines evolve gradually and smoothly, so the three 1D soliton arrays can be traced with the help of the three $\Delta\boldsymbol{u}$ directions. Secondly, the dark lines in fig. S3A become significantly wider and easier to identify with decreasing ω, so combining images at different ω allows us to identify all of the soliton-induced features and thus the locations of the solitons. Lastly, AA stacked regions can be identified from the intersections of dark lines in figs. S3A and 3B, and dark spots in fig. S3C, which form a gradually distorted hexagonal pattern throughout the entire area. The adjacent AA stacked regions are connected by solitons. Therefore, by tracing the gradual spatial evolution of soliton-induced features and the locations of AA stacked regions, the soliton network of the entire region can be determined (fig. S3D).

S2.2 Determination of 2D soliton angle maps

The local soliton angle of the three soliton arrays and hence the 2D soliton angle map $\varphi(\boldsymbol{r})$ of a region can be obtained if $\Delta\boldsymbol{u}_1$, $\Delta\boldsymbol{u}_2$ and $\Delta\boldsymbol{u}_3$ of the soliton network are determined. To achieve this, we start with $\Delta\boldsymbol{u}_1$ estimated from a region showing nearly perfect sixfold moiré pattern (for example, the left region of Fig. 1E of the main text or region 3 of fig. S3A), in which φ of the solitons are close to 90° (shear strain) to minimize the total energy. Based on this estimated $\Delta\boldsymbol{u}_1$, it is already evident from our data (figs. S3 and S4) that the line-profiles $s(x)$ depend sensitively on φ at low doping: as φ is reduced from 90° (as the soliton orientation deviates from the estimated $\Delta\boldsymbol{u}$ direction), the dip feature in $s(x)$ diminishes gradually and disappears for φ below a critical angle φ_c, so that the soliton angles satisfy:

$$\varphi_{\text{visible}} > \varphi_{\text{vanishing}} \sim \varphi_c > \varphi_{\text{vanished}} \qquad \text{(S1)}$$

where the subscripts correspond to the type of solitons that show visible, vanishing, and vanished (no) dip features in the nano-IR images, respectively. Such a behavior is reproduced from our modelling (Fig. 2C of the main text) and can be understood from the dependence of local band structure and optical conductivity on soliton angle as discussed in section S3 below. Therefore, we treat the direction of $\Delta\boldsymbol{u}_1$(or, equivalently, another $\Delta\boldsymbol{u}$) as a fitting parameter and fine-tune it to reproduce the observed dependence of the dip features described by Eq. (S1).

As an example, the soliton angle map $\varphi(\boldsymbol{r})$ for the sample in fig. S3 is determined as follows. Firstly, we can estimate the directions of $\Delta\boldsymbol{u}_1$, $\Delta\boldsymbol{u}_2$ and $\Delta\boldsymbol{u}_3$ of the soliton network based on region 3 of fig. S3A showing moiré pattern with nearly perfect sixfold symmetry. Then, we examine the φ values for two vanished solitons P_1 and P_2 in fig. S3A (labelled in fig. S3D) corresponding to φ_1 and φ_2, respectively. Based on Eq. (S1), they should satisfy: $\varphi_1 \sim \varphi_2 \lesssim \varphi_c$. We start with the estimated $\Delta\boldsymbol{u}_2$ and treat its angle with respect to the vertical axis of fig. S3D, α, as a

fitting parameter. Fig. S3G shows the changes of φ_1 and φ_2 as a function of α. Only a narrow range of α values can lead to φ values satisfying Eq. (S1), so the direction of $\Delta\boldsymbol{u}_1$ and thus the local soliton angles can be accurately determined as shown in fig. S3D. The uniqueness of the fitting result is ensured by the opposite α-dependence for φ_1 and φ_2. We have verified that the resulting soliton angle map in fig. S3D satisfies Eq. (S1) and reproduces the observed behaviors (namely, visible, vanishing or vanished dip features) for all solitons in figs. S3 A-C. The accuracy of the obtained soliton angles is estimated to be ±1°, which is mainly determined by the uncertainties in the assignments of vanishing and vanished dip features in fig. S3A.

Other soliton angle maps shown in this study are obtained using similar approaches. The accuracy of soliton angle map in Fig. 1F of the main text is estimated to be ±2° due to larger uncertainties in the assignments of vanishing and vanished soliton-induced features in fig. S4.

S2.3 Procedures for obtaining Fig. 2A of the main text

Figs. S3a and 3d reveal a direct correlation between the observed polariton interference feature with spatial variations of φ described by Eq. (S1). Combining fig. S3A and 3D allows us to obtain the $s(x)$ profile as a function of φ (Fig. 2A of the main text) using the following procedure. Firstly, a histogram of local φ values is extracted from the soliton angle map in fig. S3D, in which each bin (bar) covers a range of soliton angle, $\Delta\varphi$, with an averaged value, $\bar{\varphi}$. Next, we take line-profiles $s(x)$ (x is the local direction normal to the soliton) across solitons in fig. S3A, and group such profiles into different bins based on the local φ values. Because we focus on $s(x)$ across single (isolated) solitons in Fig. 2A, only profiles at a sufficient distance from other solitons are included in the analysis in order to avoid significant influence from other solitons. Specifically, when taking the $s(x)$ profile at a soliton location, we define a forbidden zone within the range of $\pm w_1$ ($\pm w_2$) of that location (other solitons) along x direction. We exclude the profiles at soliton locations where these forbidden zones overlap with one another. Our superposition model (section S4.3 below) simulations show that the amplitude of the dip feature at the solitons at ω=1540 cm^{-1} is not significantly affected by other solitons for $w_1+w_2 \gtrsim 40$ nm. Finally, the averaged line-profiles for each bin are obtained by averaging all line-profiles from that bin, which are then plotted as a function of the corresponding $\bar{\varphi}$, yielding Fig. 2A. The error bars represent the standard error of the averaged line-profiles in each bin. While Fig. 2A are obtained with $\Delta\varphi$=2°, the general trend of on-off switching shown in Fig. 2A doesn't depend on the value of $\Delta\varphi$.

S3 Band structure and local optical conductivity calculations for solitons in bilayer graphene

Our calculations of a single infinitely long domain wall soliton with soliton angle φ separating bilayer graphene with AB and BA stacking are based on a continuum model for bilayer graphene with a spatial dependent interlayer displacement vector $\boldsymbol{u}(x)$ (22,44,45), where x is the direction normal to the soliton. The Hamiltonian for the basis of sublattices of layers 1 and 2 (A_1, B_1, A_2, B_2), is written as (22,44,45):

$$H = \begin{bmatrix} H_0^+ & U^\dagger(x) \\ U(x) & H_0^- \end{bmatrix} \quad \text{(S2)}$$

The Dirac Hamiltonian for a single layer is (45)

$$H_0^\pm = \begin{bmatrix} \pm\frac{V}{2} & \hbar v(\xi k_x + ik_y)e^{i(\frac{\pi}{2}-\varphi)} \\ \hbar v(\xi k_x - ik_y)e^{-i(\frac{\pi}{2}-\varphi)} & \pm\frac{V}{2} \end{bmatrix} \quad \text{(S3)}$$

where $V=eV_i$ is the interlayer potential, $\xi = \pm 1$ for the K and K' valley, $\pi/2-\varphi$ denotes the angle between the armchair direction of the graphene lattice and the soliton (y direction). The interlayer coupling terms are:

$$U(x) = \frac{\gamma_1}{3}\begin{bmatrix} 1+2\cos\left(\frac{2\pi u_Y}{3a_0}\right)e^{\frac{2\pi i\xi u_X}{\sqrt{3}a_0}} & 1+2\cos\left[\frac{2\pi}{3}\left(\frac{u_Y}{a_0}+1\right)\right]e^{\frac{2\pi i\xi u_X}{\sqrt{3}a_0}} \\ 1+2\cos\left[\frac{2\pi}{3}\left(\frac{u_Y}{a_0}-1\right)\right]e^{\frac{2\pi i\xi u_X}{\sqrt{3}a_0}} & 1+2\cos\left(\frac{2\pi u_Y}{3a_0}\right)e^{\frac{2\pi i\xi u_X}{\sqrt{3}a_0}} \end{bmatrix} \quad \text{(S4)}$$

where the interlayer coupling γ_1≈0.4 eV and the interlayer displacement vector $\boldsymbol{u}(x)= u_X(x)\,\boldsymbol{e}_X+ u_Y(x)\,\boldsymbol{e}_Y$ is defined in a coordinate system where X and Y are along the zigzag and armchair directions of the graphene lattice, respectively. Here $\boldsymbol{e}_X$ and $\boldsymbol{e}_Y$ are the unit vectors along X and Y, respectively, and a_0≈0.142 nm is the length of carbon- carbon bond in graphene. Trigonal warping terms γ_3 and γ_4 have been neglected for simplicity.

In mTBG, the stacking vectors $\boldsymbol{u}$ associated with the six domains meeting at one AA site are successively rotated by π/3(Fig. 1B). Therefore, without loss of generality, we consider a soliton with an arbitrary soliton angle φ separating two domains with stacking vectors $\boldsymbol{u_1} = -\frac{\sqrt{3}}{2}a_0\boldsymbol{e_X}-\frac{1}{2}a_0\boldsymbol{e_Y}$ and $\boldsymbol{u_2} = -\frac{\sqrt{3}}{2}a_0\boldsymbol{e_X}+\frac{1}{2}a_0\boldsymbol{e_Y}$ (Fig. 1A) both defined in the X-Y coordinate system, so that the interlayer translation vector $\Delta\boldsymbol{u}$ associated with the soliton is along the Y (armchair) direction. Based on previous transmission electron microscopy studies, the distribution of $\boldsymbol{u}(x)$ across a soliton has the form (14,34): $\boldsymbol{u}(x) = \frac{2}{\pi}\arctan\left(e^{\frac{\pi x}{w}}\right)a_0\boldsymbol{e_Y} + \boldsymbol{u_1}$, where the width of the soliton w is given by ref. 14: $w = \frac{a_0}{2}\sqrt{\frac{1}{V_{sp}}\left(\frac{Et}{1-\upsilon^2}cos^2\varphi + Gtsin^2\varphi\right)}$,

V_{sp} is saddle-point energy per unit area, Et=340 N/m is the Young's modulus, υ is the Poisson ratio, $Gt \sim Et/(2(1+\upsilon))$=142 N/m is the shear modulus. The width w for the tensile (shear) wall is about 10.1 nm (6.2 nm). In the x-y coordinate system, the momentum k_y remains a good quantum number. The momentum k_x perpendicular to the soliton is replaced by the operator $-i\partial/\partial x$. The resulting real space Hamiltonian $H(x, k_y)$ is solved numerically on a 1D grid along x direction to obtain electronic band structures as shown in fig. S5 and 6. In the cases of shear and tensile walls, we obtain similar results as reported in previous studies (22).

Next, the eigenvalues (E_m, E_n) and eigenfunctions ($|u_m\rangle$, $|u_n\rangle$) of the Hamiltonian are used to compute the nonlocal optical conductivity $\Sigma(x, x')$ employing the Kubo formula (22), with inter- and intra-band contributions given by:

$$\Sigma^{\mathrm{I}}_{\alpha\alpha}(x,x') = \frac{g_s g_v i\hbar}{4\pi^2}\int dk_y \sum_{n\neq m} -\frac{f_m - f_n}{E_m - E_n}\frac{e^2 v^2 M^*_\alpha(x) M_\alpha(x')}{\hbar\omega + i\eta - (E_m - E_n)} \quad \text{(S5)}$$

$$\Sigma^{\mathrm{D}}_{\alpha\alpha}(x,x') = \frac{g_s g_v i\hbar}{4\pi^2}\int dk_y \sum_{n} -\frac{df_n}{dE_n}\frac{e^2 v^2 M^*_\alpha(x) M_\alpha(x')}{\hbar\omega + i\eta} \quad \text{(S6)}$$

where x and x' are coordinates in the direction normal to the soliton, $\alpha = x$, or y (in this work, we focus on Σ_{xx}). $g_s = g_v = 2$ denotes the spin and valley degeneracy in graphene. $f(E_m)$ is the Fermi-Dirac distribution $f(E_m) = 1/\left(1 + \mathrm{e}^{(E_m-\mu)/k_\mathrm{B}T}\right)$. The matrix element is defined as $\mathrm{M}_\alpha = \langle u_m | v_\alpha | u_n \rangle$, and the velocity operator is obtained from the Hamiltonian through $v_\alpha = \frac{\partial H}{\hbar\, \partial k_\alpha}$. The parameters in the formula are chemical potential μ, temperature T, frequency ω, interlayer bias V_i and damping rate η. Then the local optical conductivity can be obtained from $\sigma(x) \equiv \int \Sigma(x,x')\, dx'$.

The electronic structure and the local optical conductivity $\sigma(\omega, x) \equiv \sigma_{xx}(\omega, x)$ of the solitons with different soliton angles φ are shown in fig. S5 (6) for low (high) doping, which can reproduce the experimental data at V_g=0 V(V_g=–6 V). While we present $\sigma(\omega, x)$ and the simulated $s(x)$ obtained using μ=5 meV and V_i=2 mV in fig. S5 for simulations of experimental $s(x)$ at V_g=0, μ within the range of 0–25 meV produces similar φ-dependent behaviors in the simulated $s(x)$ profiles. To simulate data at V_g=–6 V (with carrier density ~6×10^{12} cm^{-2}), we calculate μ and V_i based on self-consistent tight-binding calculations following previous studies (46), from which μ and V_i as a function of carrier density are obtained for bilayer graphene with a single bottom gate. From such calculations, μ=190 meV and V_i =96 meV are obtained for the carrier density at V_g=–6 V, which are used for obtaining the $\sigma(\omega, x)$ and the simulated $s(x)$ profiles in fig. S6. We also examine the behaviors of $\sigma(\omega, x)$ and the resulting $s(x)$ profiles for larger values of V_i (for example, 300 meV) following ref. 22, as shown in fig. S6.

The electronic structure and the local optical conductivity $\sigma(\omega, x) \equiv \sigma_{xx}(\omega, x)$ of the solitons with different soliton angles φ are shown in fig. S5 (6) for low (high) doping, which can reproduce the experimental data at V_g=0 V(V_g=–6 V). A finite interlayer bias V_i opens a bandgap in the band continua of bulk AB (BA) bilayer graphene; however, topological chiral states localized at the soliton must exist across the gap due to the different valley Chern numbers associated with the AB and BA regions. While the shear solitons only host such topological states, conventional (non-topological) bound states outside the band continua also exist in non-shear solitons (fig. S5 and 6). Moreover, we find high-energy states localized at the soliton inside the band continua for all types of solitons. Both these high-energy states and the conventional bound states in non-shear solitons are inherited from the local band structure around SP stacking at the soliton and can be understood as electronic waveguide modes confined to the solitons (22). Most interestingly, the soliton band structure evolves continuously between those of the shear and tensile solitons as the soliton angle φ changes (fig. S5 and 6). Such an evolution can be qualitatively understood (in a "adiabatic" picture (22)) as the superposition of local 2D band structures $E(k_x, k_y)$ at various locations across the soliton projected to the 1D momentum axis parallel to the soliton, with the projection angle being φ. While this provides a qualitative picture for understanding the results, we use the "lattice" approach (22) described above in our calculations, which can account for all the effects.

The evolution of the band structure with φ profoundly modifies the interband transitions

involving the soliton states and hence the local optical conductivity. Optical transitions involving the conventional bound states give rise to a strong resonance peak around 0.1 eV in real part of the optical conductivity Re $\sigma(\omega)$ for the tensile wall. For the shear wall, optical transitions from topologically protected states to high-energy soliton states produce a peak around 0.22 eV in Re $\sigma(\omega)$. The resonance peaks in both cases show large enhancement compared to values at the AB domain in the same frequency, and are accompanied by a large "Lorentzian dip" in imaginary part of the optical conductivity Im $\sigma(\omega)$ lower than the value of Im $\sigma(\omega)$ for the AB stacking. We find that the $\sigma(\omega)$ and $\sigma(x)$ spectra evolve continuously from shear to tensile solitons, due to the continuous change of band structure with soliton angle. In particular, the resonance associated with the optical transition around 0.22 eV is continuously suppressed as the soliton evolves from shear to tensile, leading to the features in $\sigma(x)$ in fig. S5. The evolution of $\sigma(x)$ with φ is essentially similar for all excitation frequencies within the upper Reststrahlen band of hBN. In this Reststrahlen band, the local enhancement of Re $\sigma(x)$ at the soliton is small at low μ and becomes very large at high μ (fig. S6). The latter case mainly results from the suppression of Re $\sigma(\omega)$ at the AB region due to the Pauli blocking of interband transitions below 2μ, leading to a strong peak in Re $\sigma(\omega)$ at the soliton for all φ (fig. S6).

S4 Simulations of line-profiles, polariton field distributions and near-field images

For a quantitative analysis of the observed soliton-angle- and gate-dependent near-field contrast, numerical simulations of the near-field line-profiles $s(x)$ across a single soliton are performed using two methods: a finite-element method (47,48) and an analytical model (49), which produce consistent results. The structure in the simulations consists of bilayer graphene with a single soliton, hBN and SiO_2. The thickness of hBN is 19 nm and that of SiO_2 is 285 nm. For the top layer of the structure, $\sigma(x)$ around a soliton at various φ shown in fig. S5 and 6 are used. The dielectric function of SiO_2 is modeled using the data in ref. 50, which are interpolated with Brendel oscillators as in ref. 51. The dielectric function of hBN is a dielectric tensor with in-plane components $\varepsilon_x(\omega) = \varepsilon_y(\omega)$ and out-of-plane component $\varepsilon_z(\omega)$, which are modeled with the Lorentz model with the same parameters as in refs. 31 and 32.

S4.1 Finite-element method: simulation of line-profiles and polariton field distributions

In the finite-element method, simulations of the near-field profiles are performed using previously developed techniques based on the finite-element method (47,48) in the quasi-electrostatic limit (29), which has been shown to provide a quantitative description of near-field spectroscopy and polariton imaging (29,47,48). The tip is approximated as an elongated conducting spheroid. A constant electric field in the z direction, $E_0\hat{\mathbf{z}}$, is used as a background excitation to induce the tip-sample near-field interaction, which further modifies the charge distribution along the spheroid surface and thus the dipole moment. Due to the elongated tip shape as well as the background field direction, only the dominant dipole moment in the z direction, p_z, is considered. The distance between the spheroid and the sample surface is varied according to $dz = A(1-\cos(\Omega t)) + h_0$, where A=50 nm is the tip tapping amplitude, h_0=1 nm is the minimal tip-sample distance, and Ω is the tip tapping frequency. Finally, to obtain the near-field amplitude s_n and phase ψ_n, $p_z(t)$ is demodulated to higher harmonics of the tip oscillation frequency as

$$s_n e^{i\psi_n} = \int_0^T p_z(t) e^{in\Omega t} dt \quad \text{(S7)}$$

where n=3 and T=2π/Ω. To simulate a spatial scan, the lateral position of the tip is varied. We ensure that the step size is sufficiently small to resolve any observable features.

S4.2 Analytical model

Conceptually, near-field response from a domain wall in twisted bi-layer graphene can be calculated in the same way described in ref. 49. The model takes into account the polarization of the tip induced by sample-tip near field interactions, the oscillations of the tip and the demodulation of the near-field signal. The main difference between the present simulations is that we now consider a single 2D material's conductivity boundary instead of a periodic array. As a result, here, we deal with the Fourier transform of the desired values instead of the Fourier series. The layered wafer with anisotropic hyperbolic materials supporting TBG includes a conductivity boundary of arbitrary shape instead of a rectangular profile discussed in ref. 49. A cylindrical tip of radius a=30 nm oscillating near the sample with a minimal approaching distance of $d_{\min}$=0 nm and maximum $d_{\max}$=50 nm.

In our simulations, the near-field response is calculated from a dipole moment $\vec{p}$ of the tip averaged over its position oscillating along the normal to sample direction. The dipole moment is generated both by the electric field $\vec{E}_0$ of the incident laser beam and by the electric field $\vec{E}^{\text{ind}}$ induced by the sample due to the tip's dipole moment and its near field. As was shown in ref. 49, $\vec{E}^{\text{ind}}$ and $\vec{p}$ are related with $\hat{\beta}$-tensor as $\vec{E}^{\text{ind}} = \hat{\beta}(h)\vec{p}/2\varepsilon_0 h$, where h is the height of the tip's dipole moment above the sample and $\varepsilon_0 = 1$ is the dielectric permittivity of the medium above the sample (see fig. S11). In our calculations, we neglect the tip's polarization in x-direction (parallel to the sample), and thus beta tensor reduces to a scalar value. To find $\hat{\beta}$, we calculate $\vec{E}_x^{\text{ind}}$ for $\vec{p} = 1 \cdot \vec{\imath}$ and $\vec{E}_z^{\text{ind}}$ for $\vec{p} = 1 \cdot \vec{k}$, where $\vec{\imath}$ and $\vec{k}$ are the unity vectors in x- and z- directions, and then form the tensor from the found vectors components:

$$\hat{\beta} = 2\varepsilon_0 h \begin{pmatrix} E_{x,x}^{\text{ind}} & E_{z,x}^{\text{ind}} \\ E_{x,z}^{\text{ind}} & E_{x,z}^{\text{ind}} \end{pmatrix}. \quad \text{(S8)}$$

The calculation of $\vec{E}^{\text{ind}}(\vec{p})$ now is reduced to matching boundary conditions at the interfaces between the media with ε_0, ε_1 and ε_1, ε_2 for the given electric field generated by the dipole moment $\vec{p}$ at the ε_0, ε_1 interface. For $\vec{p} = 1 \cdot \vec{\imath}$, the induced field is given by:

$$E_{x,x}^{\text{ind}} = \frac{-2(h^2 - x^2)}{(h^2 + x^2)^2}, \quad \vec{E}_{x,z}^{\text{ind}} = \frac{-4hx}{(h^2 + x^2)^2}, \quad \text{(S9)}$$

and for for $\vec{p} = 1 \cdot \vec{k}$

$$E_{z,x}^{\text{ind}} = \frac{-4hx}{(h^2 + x^2)^2}, \quad \vec{E}_{z,z}^{\text{ind}} = \frac{2(h^2 - x^2)}{(h^2 + x^2)^2}. \quad \text{(S10)}$$

To find the basis set solutions in all three media, we solve the Poisson equation with anisotropic dielectric permittivity given as diagonal tensor $\hat{\varepsilon}_l$ with non-zero xx and zz components, where l is the media index (0, 1, or 2):

$$\nabla(\hat{\varepsilon}_l \nabla \varphi_l) = 0, \quad \text{(S11)}$$

where ∇ is the nabla operator. The solution to this equation is found using the following ansatz:

$$\varphi_{q,l}(x,z) = \varphi_{q,l}e^{-iqx\mp\varkappa_l z},\ \ \varkappa_l = \sqrt{\frac{\varepsilon_{x,l}}{\varepsilon_{z,l}}},\ \arg\varkappa_l \in \left(-\frac{\pi}{2},\frac{\pi}{2}\right], \tag{S12}$$

where $\varphi_{q,l}$ are their amplitudes in Fourier space (or Fourier transforms of $\varphi_l(x,z)$). The potential generating electric field Eq. (S9) and Eq. (S10) at the ε_0, ε_1 interface are the following: $\varphi_{q,0}^{p_x} = 2\pi\frac{iq}{|q|}e^{-h|q|}$, $\varphi_{q,0}^{p_z} = -2\pi e^{-h|q|}$. Although these waves are evanescent, one can introduce reflection and transmission coefficients for them at the media interface and calculate their scattering in planar structures using transfer matrices as it used to do in the case of propagating waves. Thus, an evanescent wave "falls" at an interface ε_k, ε_l with the sign "$-$" before $\varkappa_k$, reflects back to the medium k with the sign "+", and passes into the medium l with the sign "$-$" before the evanescent wave parameter $\varkappa_l$ (fig. S11). The reflections coefficients are the following:

$$r_{kl} = \frac{\varepsilon_{x,l}\varkappa_k - \varepsilon_{x,k}\varkappa_l}{\varepsilon_{x,l}\varkappa_k + \varepsilon_{x,k}\varkappa_l},\quad t_{kl} = \frac{2\varepsilon_{x,l}\varkappa_k}{\varepsilon_{x,l}\varkappa_k + \varepsilon_{x,k}\varkappa_l}. \tag{S13}$$

To find the sample back-action on the s-SNOM tip's dipole, we need to solve together the following equations:

• Boundary conditions at the 2D material plane (here and below, we omit "0" sub-index in $\varphi_{q,l}$ for $l = 0$):

$$\varphi_q(1+\rho_q)iq = E_{x,1q}, \tag{S14}$$

$$\varphi_q(-1+\rho_q)\varepsilon_{z,0}\varkappa_0 - D_{z,1q} = 4\pi\sigma_q, \tag{S15}$$

where $E_{x,1q}$ and $D_{z,1q}$ are the Fourier transforms of the electric field's tangential to the sample plane projection and of the electrical displacement vector's normal projection in ε_1 medium in the vicinity to the sample; and σ_q is the Fourier transform of the electric charge density (per unit of area) induced in the 2D material.

• Found with transfer matrix formalism relation $D_{z,1q}$ to $E_{x,1q}$:

$$\frac{D_{z,1q}}{E_{x,1q}} = i\frac{\varepsilon_{z,1}\varkappa_1}{q}\frac{1-r_{12}e^{-2\varkappa_1 h}}{1+r_{12}e^{-2\varkappa_1 h}}. \tag{S16}$$

• Ohm's law for the 2D material (in real space) $E_{x,1}(x) = \rho(x)j(x)$ (where $\rho(x)$ is the electrical resistivity of the 2D material and $j(x)$ is the electrical current through the 2D material per unit of its length transverse direction), what in the Fourier space can be rewritten as:

$$E_{x,1q} = \rho_0 u_q + \frac{1}{2\pi}\int_{-\infty}^{\infty}\delta\rho_{q-p}u_p dp, \tag{S17}$$

where ρ is represented as the sum of two terms $\rho(x) = \rho_0 + \delta\rho(x)$, $\delta\rho(\pm\infty) = 0$, u_q and $\delta\rho_q$ are the Fourier transforms of $j(x)$ and $\delta\rho(x)$:

$$u_q = \int_{-\infty}^{\infty} j(x)e^{iqx}dx,\quad \delta\rho_q = \int_{-\infty}^{\infty}\delta\rho(x)e^{iqx}dx. \tag{S18}$$

Combining the Eqs (S13)–(S16), we obtain the relation between $E_{x,1q}$, u_q and φ_q:

$$\frac{2E_{x,1q}}{iq}\frac{1+r_{01}r_{12}f_1}{(1+r_{01})(1+r_{12}f_1)} - \frac{4\pi}{\omega}\frac{qu_q}{\varepsilon_{z,0}\varkappa_0} = 2\varphi_q, \tag{S19}$$

where $f_1 = e^{-2\varkappa_1 h}$. Substituting Eq. (S17) into the recent equation, we obtain:

$$\left(\rho_0 - \frac{2\pi i}{\omega}\frac{q^2 u_q}{\varepsilon_{z,0}\varkappa_0}\frac{(1+r_{01})(1+r_{12}f_1)}{1+r_{01}r_{12}f_1}\right)u_q + \frac{1}{2\pi}\int_{-\infty}^{\infty}\delta\rho_{q-p}u_p dp = \frac{(1+r_{01})(1+r_{12}f_1)}{1+r_{01}r_{12}f_1} iq\varphi_q \quad \text{(S20)}$$

After discretizing the integral Eq. (S20), one can reduce this integral equation to the system of linear algebraic equations, which can be solved numerically. Then, after u_q are known, one can calculate the amplitude of the "reflected" near field $r_q\varphi_q$ with Eq. (S14) and then using Eq. (S12) calculate $E_x^{\text{ind}}$and E_z^{ind} as:

$$\begin{aligned} E_x^{\text{ind}} &= \frac{1}{2\pi}\int_{-\infty}^{\infty} iqr_q\varphi_q e^{-\varkappa_l h}dq, \\ E_z^{\text{ind}} &= \frac{1}{2\pi}\int_{-\infty}^{\infty} \varkappa_l r_q\varphi_q e^{-\varkappa_l h}dq. \end{aligned} \quad \text{(S21)}$$

These values form $\hat{\beta}$-tensor (see Eq. (S8)), which is used for the calculation of near-field response as described in detail in ref. 49.

S4.3 Simulation of near-field images using a superposition model

The near-field images resulting from polariton interference induced by a soliton network can be analyzed using a superposition model (21), which neglects any soliton interactions at their intersections as well as multiple scattering of polaritons by the solitons. The soliton network consists of three 1D soliton arrays rotated by certain angles in-plane with respect to each other. Within this model, the complex near-field signal produced by one such array with parallel solitons located at positions x_k is given by:

$$\sum_k s(x - x_k)\, e^{i\psi(x-x_k)} \quad \text{(S22)}$$

in which polariton interference effects induced by neighboring solitons are explicitly taken into account. Here $s(x)$ and $\psi(x)$ are near-field amplitude and phase profiles for a single isolated soliton, respectively, and x is the direction normal to the soliton array. The signals from the other two 1D arrays are calculated similarly. Due to complications discussed in section S4.4, experimental $s(x)$ and $\psi(x)$ profiles are used in our simulation using the superposition model.

Simulations from this model show excellent agreement with experimental data, as demonstrated in previous studies (21) and our own study. The simulated images displayed in fig. S3E and 3F are produced by the superposition of signals from one and three soliton arrays, respectively, using the experimental $s(x)$ and $\psi(x)$ profiles acquired at V_g= 0 V with ω=1550 cm^{-1} as inputs. Moreover, the experimental image (V_g=–6 V) shown in Fig. 4C of the main text can be well reproduced by simulated $s(\omega)$ image (fig. S8C) obtained by superposition of signals from three soliton arrays in a distorted lattice (fig. S8B) using experimental profiles acquired at ω=1530 cm^{-1} and V_g=–6 V as inputs.

S4.4 Explanatory descriptions of the simulated results

In the literature, different phonon frequency and phonon broadening parameters are reported for hBN (32,52) and SiO_2 (24,29,50), possibly due to differences in samples and losses in thin films (52). Using different parameter sets for the dielectric functions of hBN and SiO_2 changes the widths of the simulated $s(x)$ profiles, but doesn't affect their φ-dependence, which attests to the robustness of the main results of our analysis. Therefore, we present the simulated $s(x, \varphi)$ profiles in normalized coordinate x/W, which doesn't depend on the parameters of hBN and SiO_2 used in the simulation. Here W is a width beyond which the simulated profiles become flat at low doping, which is 100-150 nm for typical parameters.

In simulations using both finite-element and analytical methods, the simulated $s(x,\ \varphi)$ profiles captures the experimental results very well, but show different lineshapes compared to experimental results in the range of x/W<0.16 (x<25 nm). This can be attributed to the assumptions and limitations of these methods. In the finite-element simulation, the results depend on the diameter of the tip in the simulation. A cylindrical tip is used in the analytical model, so it fails to reproduce the experimental results for very small x. Nevertheless, both finite-element and analytical methods can successfully reproduce the φ- and gating-dependence of the experimental $s(x)$ profiles, which is the main finding of our study. For this reason, the simulated $s(x)$ profiles are shown in the range of x/W>0.16 in figs. S5 and 6.

The experimental $s(x)$ profiles can be well reproduced by our simulations using the parameters shown in figs. S5 and 6. At low doping (μ), while $s(x)$ for solitons near shear strain exhibits a pronounced polariton interference pattern, the interference fringes are significantly reduced and vanishes for φ<75° (Fig. 2C of the main text and figs. S5 G and J). In the simulated $s(x)$, the interference fringes reappear as φ decreases further (φ<60°), but with a phase shift of π (fig. S5J). The strong φ-dependent near-field profiles at low doping mainly originate from the significant φ-dependence of Im $\sigma(x)$ (fig. S5), since the change of Re $\sigma(x)$ with φ is very weak. At high doping, on the other hand, our calculations show that the strong resonance peak at the soliton in Re $\sigma(x)$ (fig. S6I) plays a dominant role in leading to strong interference features in the $s(x)$ profiles for all φ (figs. S6 G, H, K, L), namely, all three arrays of solitons in the network. A comparison between the results for V_i=96 mV and those for V_i=300 mV shows that an increase in V_i leads to higher signal strength at the soliton.

The simulated $s(x)$ profiles displayed in Fig. 2C of the main text and figs. S5 and 6, especially their φ-dependence, are robust results and insensitive to the choice of parameters in the simulations. We use μ =5 meV and V_i=2 mV for the simulations of $s(x)$ at V_g =0. For such simulations at low doping, μ within the range of 0–25 meV produces similar behaviors in the simulated $s(x)$ profiles. Decreasing the damping rate η increases the amplitude of the features in simulated $s(x)$ profiles. Moreover, a change to the interlayer coupling γ_1 varies the contrast between Im $\sigma(x)$ and thus the simulated $s(x)$ at various φ at low doping, but these changes do not alter the φ-dependence of the simulated $s(x)$. The $\sigma(\omega)$ spectra shown in the normalized frequency ω/γ_1 (figs. S5E and 5F) are independent of the value of γ_1, so changes in the γ_1 parameter shifts the absolute frequency of the dip feature in Im $\sigma(x)$ at low doping, changing the degree of contrast between Im $\sigma(x)$ at various φ at a fix frequency (excitation frequency ω in the experiments).

S5 Extended results demonstrating polariton manipulation by soliton angle profiles

S5.1 Tuning polariton-soliton interactions with φ at high doping

Fig. S7 shows the φ-dependent optical conductivity profiles $\sigma(x)$ at high doping at a representative frequency, 645 cm^{-1}. For shear solitons (φ=90°), both real and imaginary parts of $\sigma(x)$ are nearly flat, corresponding to very weak polariton-soliton interactions ("OFF" state). With decreasing φ, a very strong resonance peak develops in the real part of $\sigma(x)$, indicating strong polariton-soliton interactions ("ON" state) as discussed in previous sections and figs. S5 and 6.

Such behaviors are caused by the dramatic φ–dependence of a resonance around 0.1 eV in $\sigma(\omega)$ (figs. S6E and 6F). Similarly, soliton angle dramatically affects the polariton-soliton interactions in the range of 650-900 cm^{-1} at high doping, which is relevant for manipulating polaritons in 2D materials such as mTBG, hBN, α-MoO_3 using heterostructures consisting of mTBG. In this frequency range, high doping can lead to longer polariton propagation length (lower losses) compared to the case of low doping.

S5.2 Spatial tuning of the propagation direction of polaritons

Our data show that directional polaritons can travel parallel, perpendicular or oblique to a boundary (fig. S9) that separates two regions featuring directional (region 1) and hexagonal (region 2) polariton wavefronts. Such behaviors arise from different orientations of the shear solitons in region 1, which govern the propagation of directional polaritons (Fig. 4 of the main text) with respect to the boundary. These findings indicate that the directional polaritons could be steered to different directions by designing and manipulating the orientations of the shear solitons in region 1, which are becoming possible due to recent experimental advances (7,18,19).

S5.3 Continuous phase tuning with soliton angle

We simulated the plasmon interference profiles (near-field amplitude profiles) produced by the solitons in mTBG using the methods discussed in previous sections. The phase θ_r of the plasmon reflection coefficient $r = |r|e^{i\theta_r}$ at the solitons dictates the position of the extrema of the interference pattern $\sim \cos(2q_p|x| + \theta_r + \theta_t)$, where q_p is the plasmon momentum and $\theta_t \approx -\pi/2$ is the tip-dependent phase shift (22). From the position of the interference extrema (figs. 10 A-F), the reflection phase θ_r can be obtained. Our simulations show that varying the soliton angle can continuously tune θ_r (fig. 10G). Therefore, the soliton angle profile $\varphi(\boldsymbol{r})$ can be potentially used for spatial control of the phase of plasmon polaritons in mTBG.

S6 Extended data from a typical mTBG/hBN/SiO_2/Si sample

To rule out possible contributions from the bottom graphite gate, we present data from a typical mTBG/hBN/SiO_2/Si sample without graphite gate in fig. S12. In such samples, soliton superlattices in mTBG can be regularly observed as shown by a typical nano-IR image in fig. S12B, in which the soliton network manifests as a triangular network of dark lines. Figs. S12D to S12F depict the nano-IR images of soliton superlattices in an adjacent region acquired at three frequencies, from which the local soliton angle map can be determined following the methods detailed in sections S2.1 and S2.2 of the Supplementary Materials. On-off switching of polariton-soliton interactions with φ can be observed as shown in fig. S12H. Moreover, in fig. S12F the on-off switching behavior manifests as the spatial evolution of strip features (two directions off) in the upper left region into hexagonal patterns (all three directions on) in the center and lower left regions, due to spatial variations of φ. Overall, the solitons with φ~70° shown in fig. S12G are in the OFF state in figs. S12D to S12F. These data confirm that all our observations are intrinsic to mTBG.

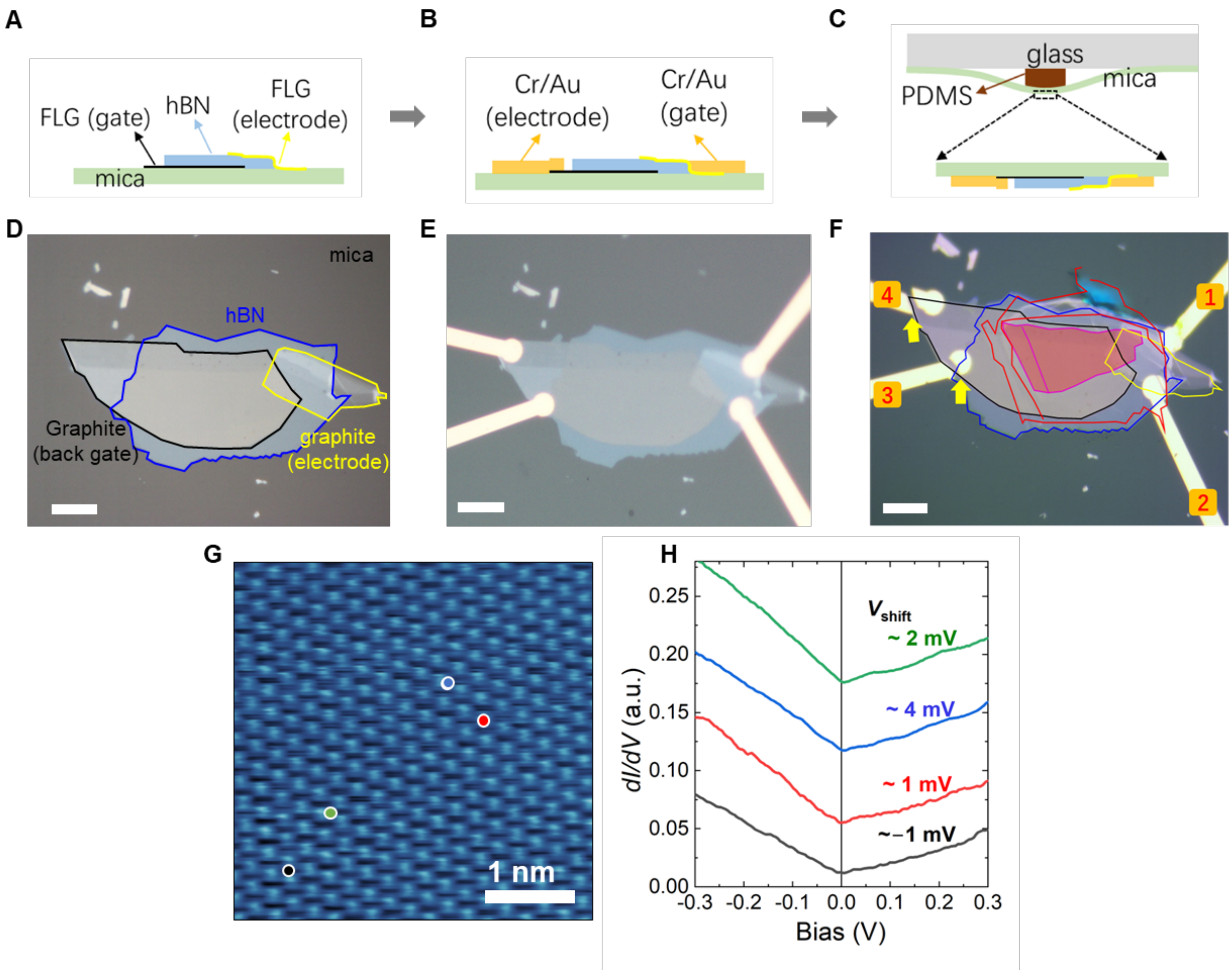

Fig. S1. Device fabrication process and characterization of the surface quality. (**A**) and (**D**), Cross-sectional schematic diagram and optical image of FLG/hBN/FLG on mica substrate after dry transfer method using PC/PDMS stamps. (**B**) and (**E**), Cross-sectional schematic diagram and optical image of FLG/hBN/FLG on mica after patterning metal leads. (**C**), Schematic diagram of mica/PDMS/glass stamp. (**F**), Optical image of the final device. Graphene layers forming TBG are identified in the area enclosed by the red lines. Scale bars in (**D**)-(**F**) are 30 μm. (**G**) and (**H**), Scanning tunneling microscopy (STM) topographic image and scanning tunneling spectroscopy (STS) obtained on the monolayer graphene region in one of the SNOM devices, showing the high-quality surface of the SNOM devices with low intrinsic doping.

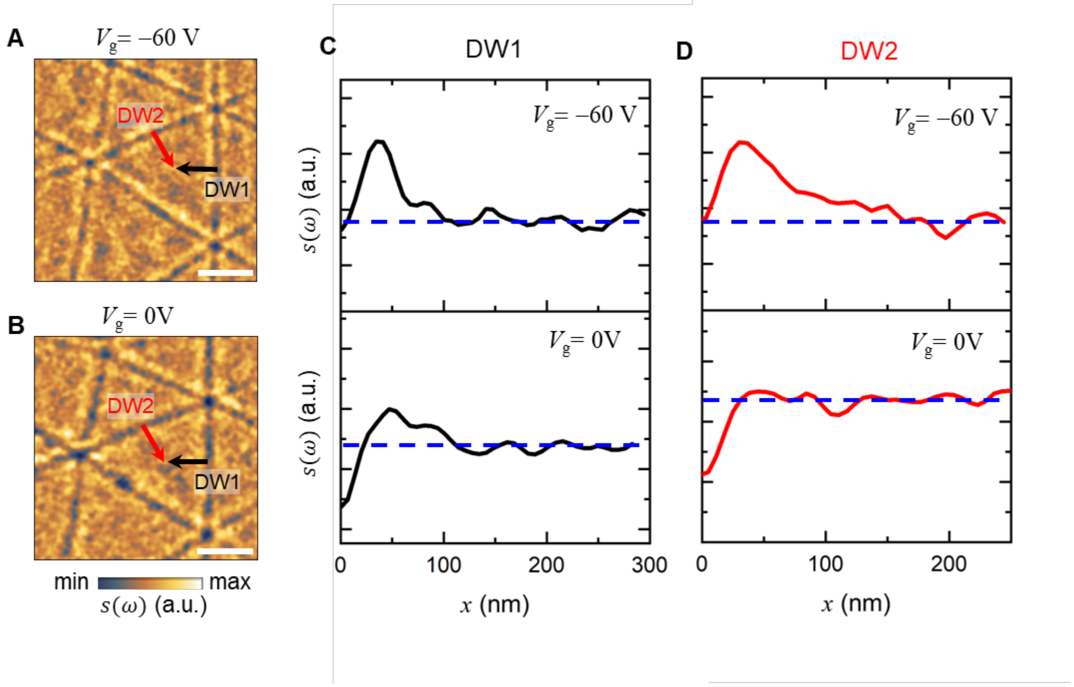

Fig. S2. Gate dependence of the near-field profile $s(x)$ across single solitons with φ~90°. (**A**) and (**B**), Nano-IR images of a nearly perfect sixfold moiré pattern in mTBG/hBN heterostructures featuring solitons with φ~90° acquired at ω=1550 cm^{-1} and two gate voltages V_g, where 285-nm-thick SiO_2 was used as the gate dielectric. Scale bars, 200 nm. (**C**)and **(D)**, Half of the near-field profile $s(x)$ across solitons (at x=0) along directions marked by the arrows at DW1 and DW2 in (**A**) and (**B**). The blue dashed lines indicate the values in the region with AB (BA) stacking. The polariton interference feature evolves from a double-peak (−60 V) to a dip feature (0 V) with decreasing V_g.

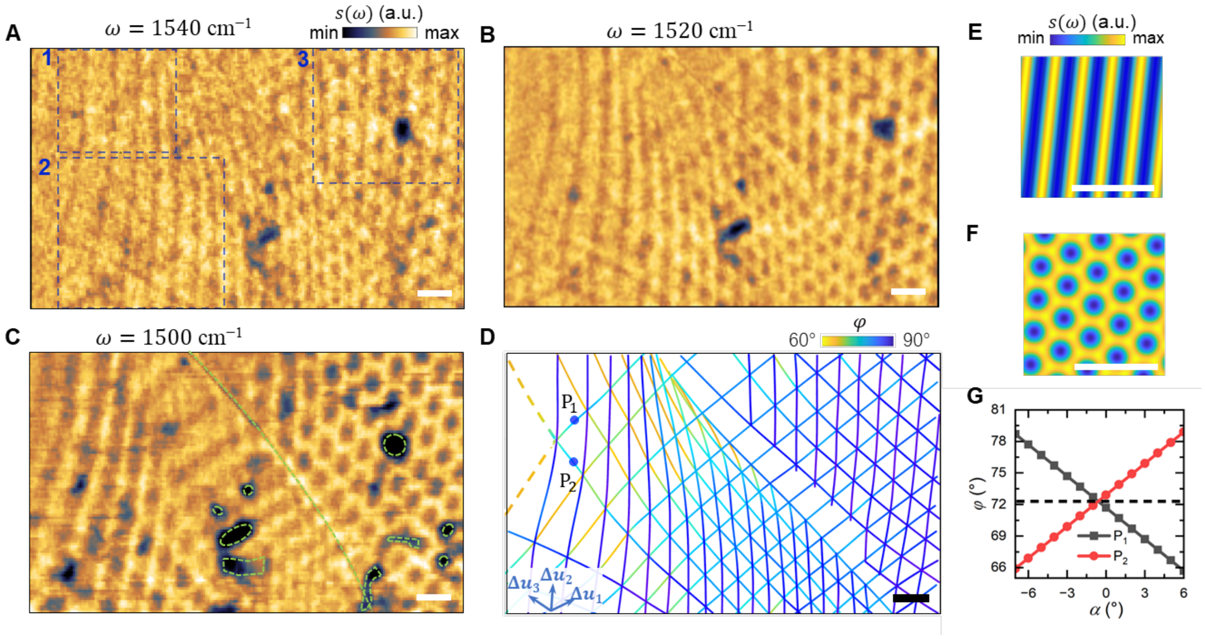

Fig. S3. Nano-IR images of a typical sample (device B) with gradient soliton superlattices. (**A**)-(**D**), Nano-IR images acquired at V_g=0 V with ω=1540 cm^{-1}. (**A**),ω=1520 cm^{-1}(**B**), ω=1500 cm^{-1} (**C**) and the corresponding local soliton angle map (**D**) for the region. Fig. 4B of the main text is the upper region in (**C**). (**E**) and (**F**), Simulated $s(\omega)$ images by the superposition model for one and three soliton arrays, respectively, with a soliton spacing of 70 nm, using the experimental $s(x)$ and $\psi(x)$ profiles across single solitons acquired at ω=1550 cm^{-1} and V_g=0 as inputs. (**E**) and (**F**) reproduce the features in the upper left and upper right regions in (**C**), respectively. (**G**), Soliton angles for two vanished solitons P_1 and P_2 labelled in (**D**) as a function of α, which is the angle of $\mathbf{\Delta u}_2$ with respect to the vertical axis of (**D**). Scale bars, 200 nm.

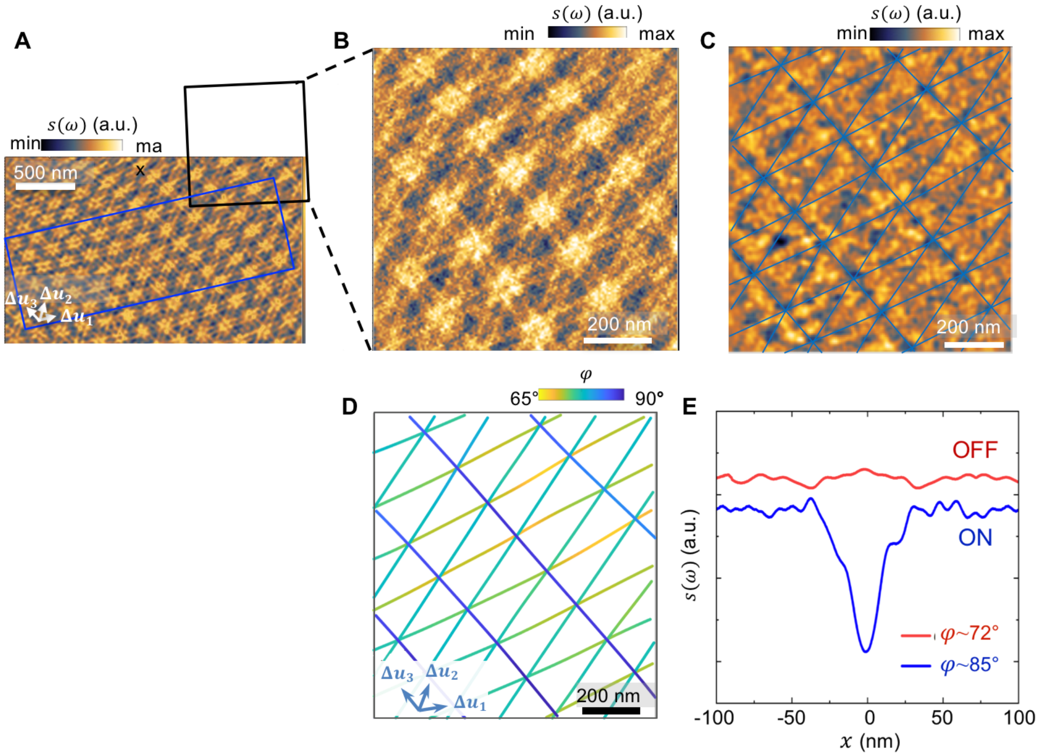

Fig. S4. Extended data showing the φ dependence of polariton interference feature at V_g=0 V. (**A**), Nano-IR image acquired at ω=1530 cm^{-1} and V_g=–6 V for device A including the region in Fig. 1E shown by the blue box. The image verifies the presence of all three soliton arrays. (**B**), Nano-IR image acquired at ω=1530 cm^{-1} and V_g=–6 V for the region in the black box in (**A**). (**C**)and (**D**), Nano-IR image (**C**) acquired at ω=1500 cm^{-1} and V_g=0 and the corresponding local soliton angle map (**D**) for the region in the black box in (**A**). The soliton network determined from (**A**)is shown as thin blue lines overlaid on the data in (**C**). (**C**)and (**D**) reveal a direct correlation between the observed polariton interference pattern with spatial variations of φ: such patterns (dark lines and segments in (**C**)) are observed at solitons with large φ, but are absent at solitons with φ≲72° in the image. (**E**), The polariton interference profiles (with the soliton at x=0) for device A extracted from (**C**)), exhibiting the on-off switching behavior with φ at low doping.

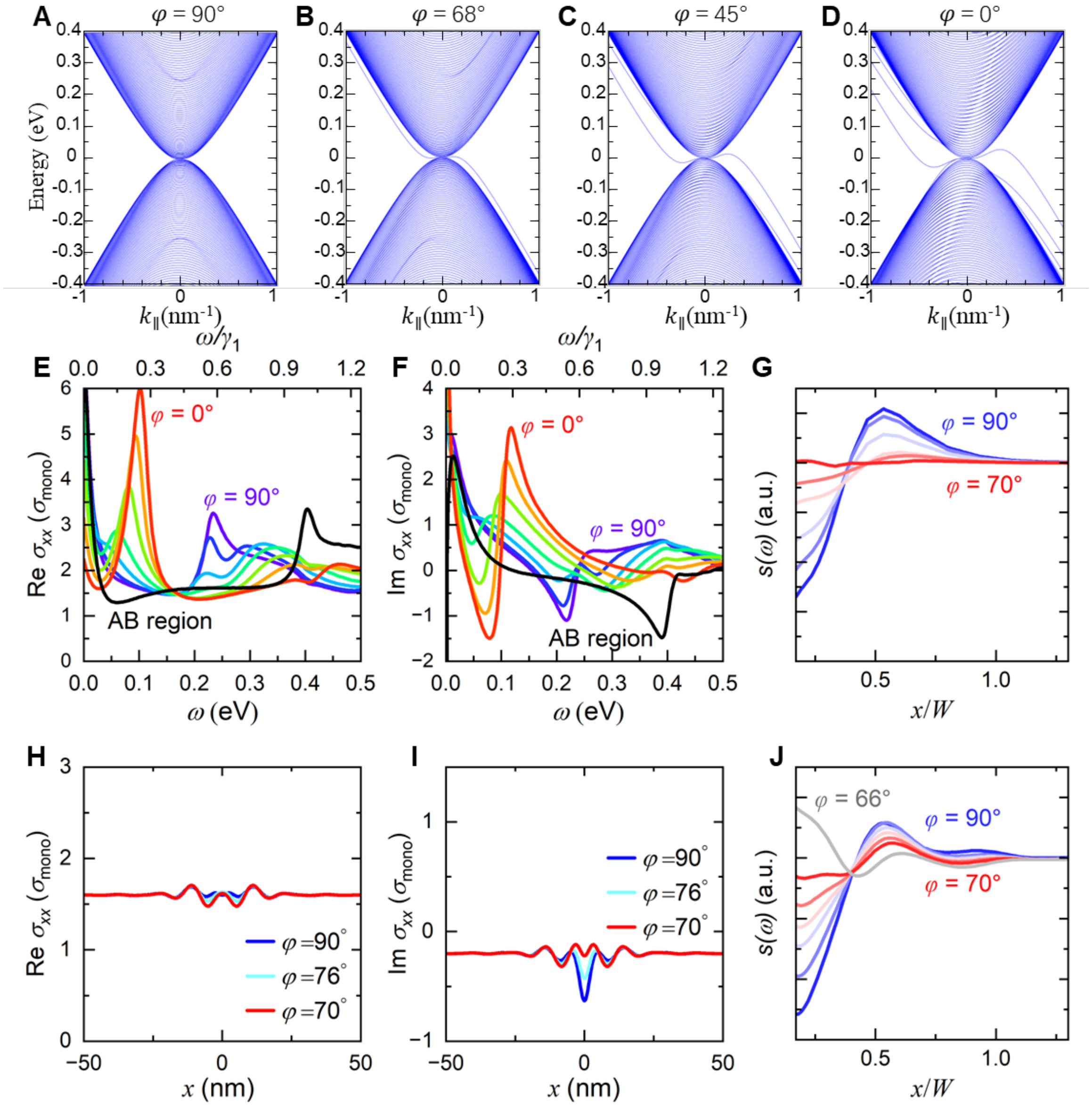

Fig. S5. Band structure, optical conductivity and simulated $s(x)$ profiles at low doping. (**A**)-(**D**), Electronic band structure of a soliton in bilayer graphene for representative soliton angles. Dispersing branches in darker colors are corresponding to electronic states with higher density of states, highlighting the high-energy soliton states (above 0.23 eV and below –0.23 eV) inside the band continua of the bulk. (**E**) and (**F**)**,** Real and imaginary parts of the local optical conductivity spectra $\sigma(\omega)$ (also shown in normalized frequency ω/γ_1) for the AB region and at the location of solitons for φ=0°, 30°, 45°, 60°, 70°, 80°, 90°. (**H**) and (**I**)**,** Spatial line-profiles of real and imaginary parts of the optical conductivity $\sigma(x)$ for ω=1540 cm^{-1} for representative soliton angles, with solitons at x=0. (**G**)**,** Simulated $s(x)$ profiles for ω=1540 cm^{-1} obtained from the finite element method for φ=70°, 71°, 72°, 76°, 81°, 90°. (**J**)**,** Simulated $s(x)$ profiles for ω=1540 cm^{-1} obtained from the analytical model for φ=66°, 70°, 72°, 74°, 76°, 80°, 90°. The simulated $s(x, \varphi)$ profiles with solitons at x=0 are shown in normalized coordinate x/W, where W is a width

beyond which the simulated profiles become flat at low doping. Parameters used for the calculations in (**A**)-(**J**) are: μ=5 meV, V_i=2 meV, η=15 meV, γ_1 =0.402 eV.

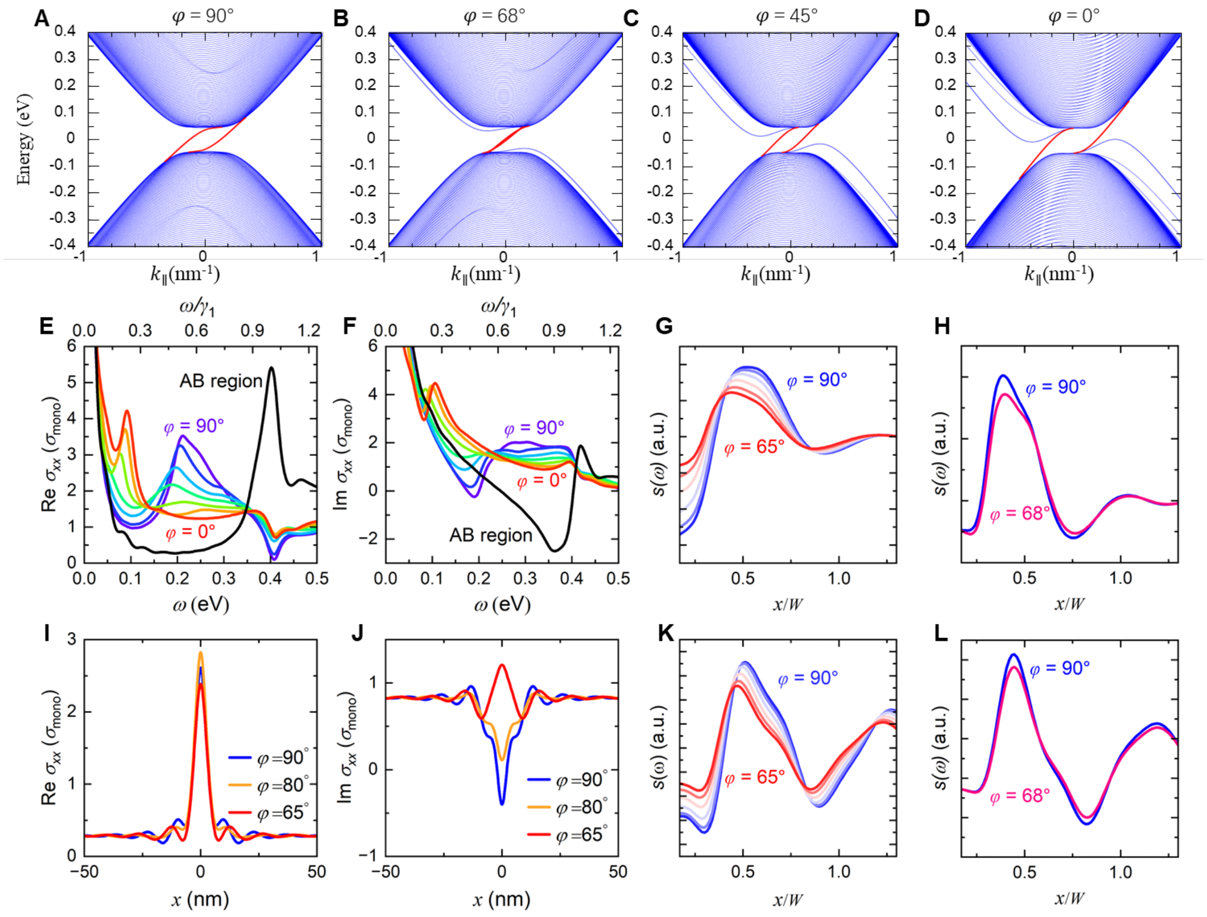

Fig. S6. Band structure, optical conductivity and simulated *s*(*x*) profiles at high doping. (**A**)-(**D**), Electronic band structure of a soliton in bilayer graphene at representative soliton angles. Dispersing branches in darker colors are corresponding to electronic states with higher density of states. (**E**), (**F**), Real and imaginary parts of the local optical conductivity spectra $\sigma(\omega)$ (also shown in normalized frequency ω/γ_1) for the AB region and at the location of solitons for φ=0°, 30°, 45°, 60°, 70°, 80°, 90°. (**I**), (**J**), Spatial line-profiles of real and imaginary parts of the optical conductivity $\sigma(x)$ at ω=1540 cm^{-1} for representative soliton angles, with solitons at x=0. (**G**), (**K**), Simulated $s(x)$ profiles for ω=1540 cm^{-1} obtained from the finite-element method (**G**) and the analytical model (**K**) for φ=65°, 70°, 75°, 80°, 85°, 90°. (**H**), (**L**), Simulated $s(x)$ profiles for ω=1540 cm^{-1} obtained from the finite-element method (**H**) and the analytical model (**L**) for a large value of V_i, V_i=300 meV; for all other panels, V_i=96 meV. Parameters used for the calculations are: μ=190 meV, η=15 meV, γ_1=0.402 eV.

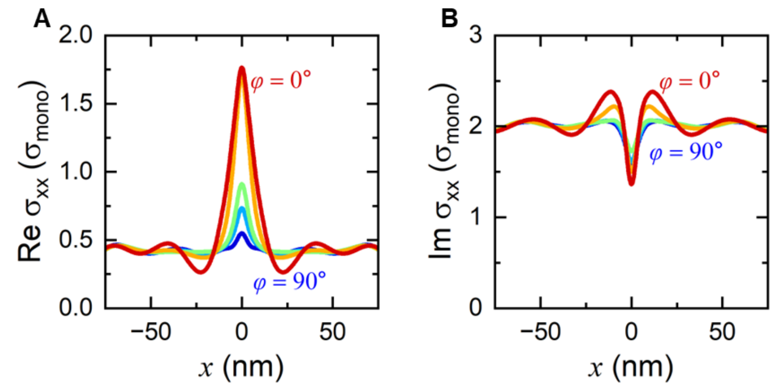

Fig. S7. Switching on/off polariton-soliton interactions with φ at high doping. (**A**), (**B**), Spatial line-profiles of real and imaginary parts of the optical conductivity $\sigma(x)$ for ω=645 cm^{-1} for representative soliton angles φ=0°, 30°, 60°, 70°, 90°, with solitons at x=0.The weak polariton-soliton interactions for φ=90° ("OFF" state) are gradually switched on as φ evolves to φ=0°. Parameters used for the calculations are: μ =190 meV, V_i =96 meV, η =15 meV, γ_1 =0.402 eV.

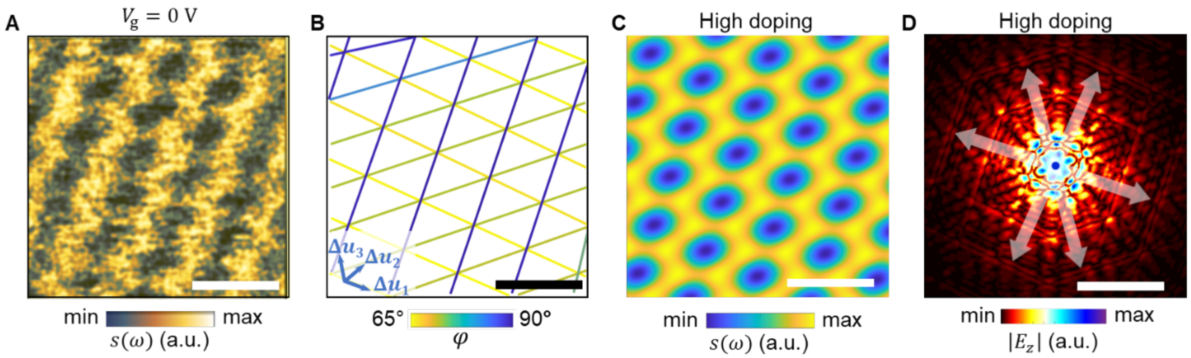

Fig. S8. Extended data and simulation results for device C. (**A**), (**B**), Nano-IR image acquired at ω=1530 cm^{-1} and V_g=0 (**A**) and the local soliton angle map (**B**) for device C shown in Fig. 4C of the main text. (**C**), Simulated $s(\omega)$ image by the superposition model for three soliton arrays with a soliton spacing of 60 nm, using the experimental $s(x)$ and $\psi(x)$ profiles across single solitons acquired at ω=1530 cm^{-1} and V_g=−6 V as inputs. (**D**), Simulated field distributions (absolute value of the z-component of the electric field $|E_z|$) of polaritons in this region at high doping, illustrating polariton propagation under the influence of three arrays of solitons in a distorted lattice, with the arrows indicating the predominant propagation directions of polaritons. Scale bars, 100 nm.

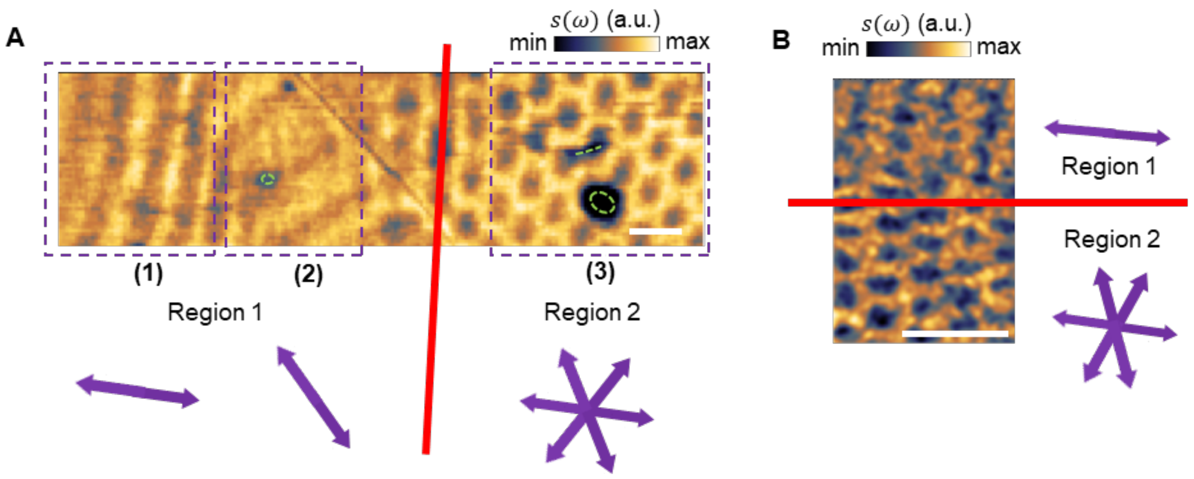

Fig. S9. Spatial tuning of the propagation direction of polaritons. (**A**), (**B**), Nano-IR images acquired at ω=1500 cm^{-1} and V_g=0 for device B (**A**) and ω=1530 cm^{-1} and V_g=–6 V for device D (**B**), both showing two regions featuring directional (region 1) and hexagonal (region 2) polariton wavefronts. The boundaries separating the two regions are shown by the red lines. The dark blue arrows indicate the predominant propagation directions of polaritons in various areas. In (**A**), directional polaritons travel perpendicular (area (1)) or oblique (area (2)) to the boundary, whereas such directional propagation is parallel to the boundary in (**B**). Scale bars, 200 nm.

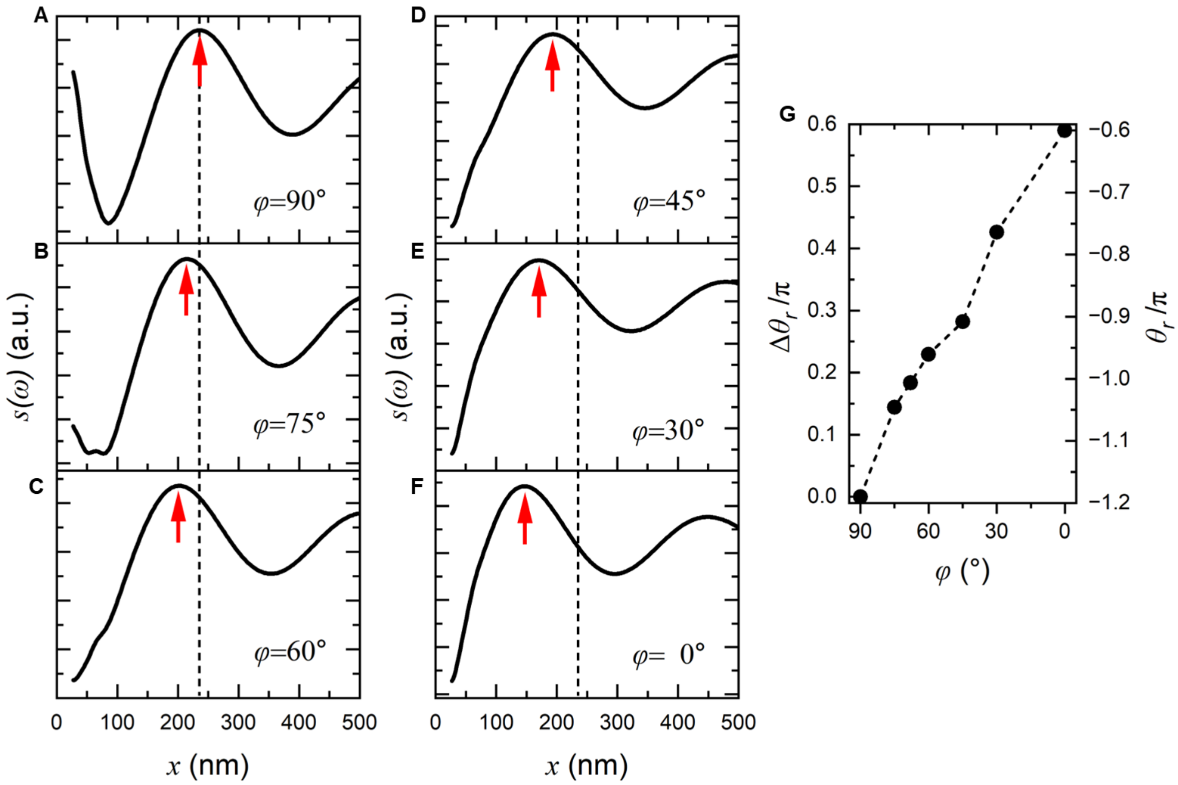

Fig. S10. Continuous phase tuning with soliton angle. (**A**)-(**F**), Simulated $s(x)$ profiles with solitons at x=0 for ω=887 cm^{-1} obtained from the analytical model for representative soliton angles. The positions of the extrema of the interference pattern are marked by red arrows. (**G**), The phase θ_r of plasmon reflection and the relative phase shift $\Delta\theta_r$ as a function of φ, where $\Delta\theta_r$ is the difference between θ_r at various φ and that at φ=90°. Parameters used for the calculations are: μ =190 meV, V_i=96 meV, η =10 meV, γ_1 =0.402 eV.

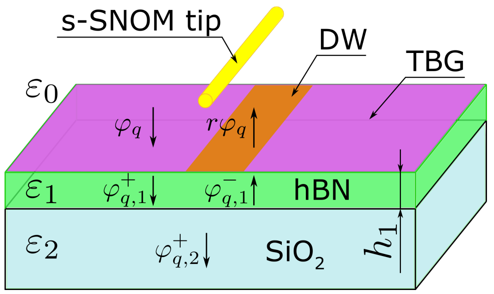

Fig. S11. Schematics of near-field simulations used in the analytical model.

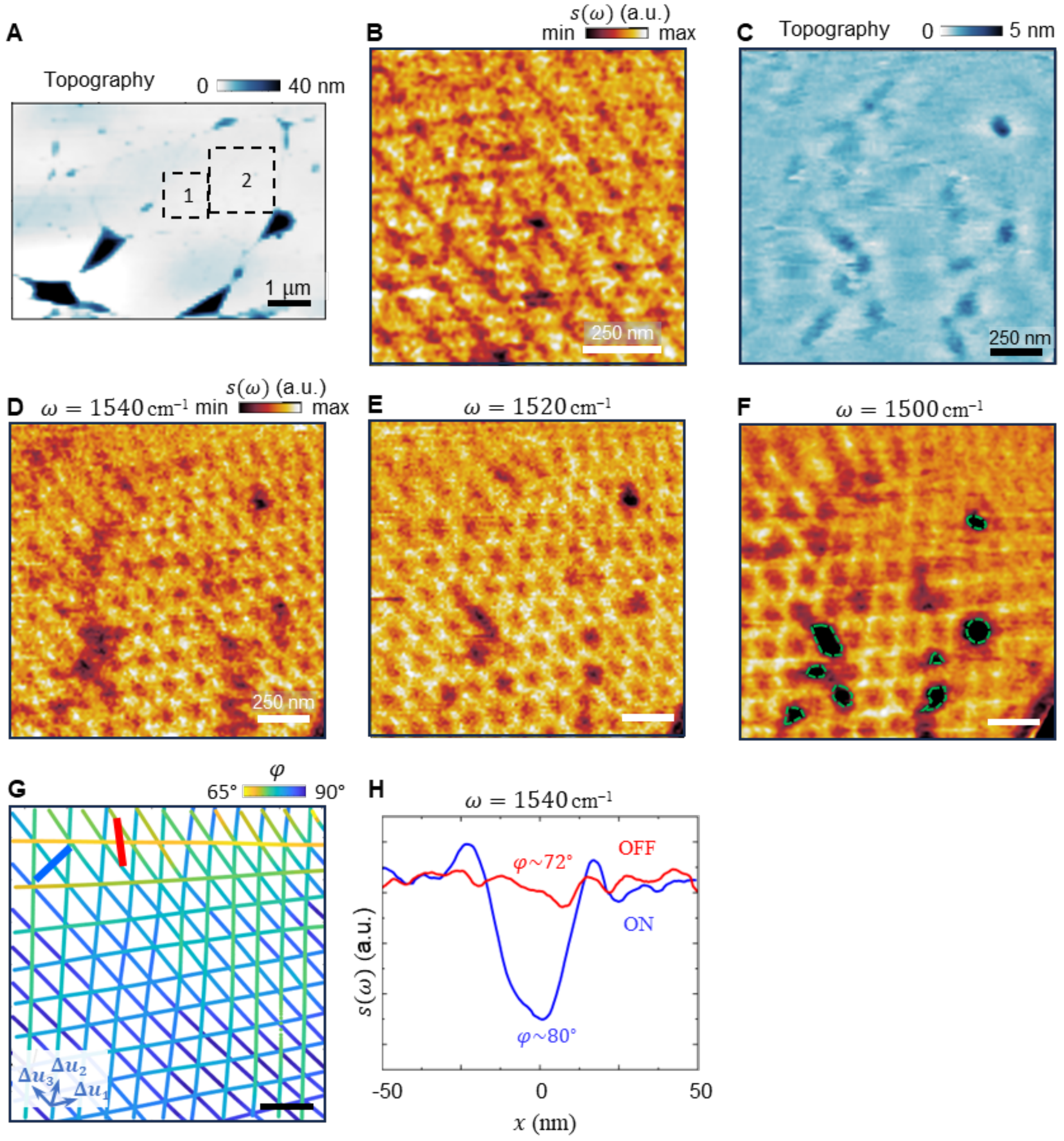

Fig. S12. Extended data from a typical mTBG/hBN/SiO_2/Si sample. (**A**), AFM topography image of a large area of the sample. (**B**), Nano-IR image acquired at ω=1540 cm^{-1} in region 1 marked in (**A**). (**C-G**), AFM topography image (**C**), nano-IR images (**D-F**) and the local soliton angle map (**G**) for region 2 marked in (**A**). Scale bars in (**C-G**), 250 nm. Locations of defects determined from (**C**) are indicated by dashed circles in (**F**). All nano-IR images were acquired at low doping. (**H**), The polariton interference profiles (with the soliton at x=0) extracted from the upper left region in (**D**) at locations marked as blue and red crosslines in (**G**), exhibiting the on-off switching of polariton-soliton interactions with φ.

REFERENCES AND NOTES

1. L. Balents, C. R. Dean, D. K. Efetov, A. F. Young, Superconductivity and strong correlations in moiré flat bands. *Nat. Phys.* **16**, 725–733 (2020).

2. D. M. Kennes, M. Claassen, L. D. Xian, A. Georges, A. J. Millis, J. Hone, C. R. Dean, D. N. Basov, A. N. Pasupathy, A. Rubio, Moiré heterostructures as a condensed-matter quantum simulator. *Nat. Phys.* **17**, 155–163 (2021).

3. E. Y. Andrei, A. H. MacDonald, Graphene bilayers with a twist. *Nat. Mater.* **19**, 1265–1275 (2020).

4. N. P. Wilson, W. Yao, J. Shan, X. Xu, Excitons and emergent quantum phenomena in stacked 2D semiconductors. *Nature* **599**, 383–392 (2021).

5. K. F. Mak, J. Shan, Semiconductor moiré materials. *Nat. Nanotechnol.* **17**, 686–695 (2022).

6. D. Zhai, W. Yao, Layer pseudospin dynamics and genuine non-Abelian Berry phase in inhomogeneously strained moiré pattern. *Phys. Rev. Lett.* **125**, 266404 (2020).

7. M. Kapfer, B. S. Jessen, M. E. Eisele, M. Fu, D. R. Danielsen, T. P. Darlington, S. L. Moore, N. R. Finney, A. Marchese, V. Hsieh, P. Majchrzak, Z. Jiang, D. Biswas, P. Dudin, J. Avila, K. Watanabe, T. Taniguchi, S. Ulstrup, P. Bøggild, P. J. Schuck, D. N. Basov, J. Hone, C. R. Dean, Programming twist angle and strain profiles in 2D materials. *Science* **381**, 677–681 (2023).

8. D. N. Basov, M. M. Fogler, F. J. G. de Abajo, Polaritons in van der Waals materials. *Science* **354**, aag1992 (2016).

9. T. Low, A. Chaves, J. D. Caldwell, A. Kumar, N. X. Fang, P. Avouris, T. F. Heinz, F. Guinea, L. Martin-Moreno, F. Koppens, Polaritons in layered two-dimensional materials. *Nat. Mater.* **16**, 182–194 (2017).

10. Q. Zhang, G. Hu, W. Ma, P. Li, A. Krasnok, R. Hillenbrand, A. Alù, C.-W. Qiu, Interface nano-optics with van der Waals polaritons. *Nature* **597**, 187–195 (2021).

11. A. Reserbat-Plantey, I. Epstein, I. Torre, A. T. Costa, P. A. D. Gonçalves, N. A. Mortensen, M. Polini, J. C. W. Song, N. M. R. Peres, F. H. L. Koppens, Quantum nanophotonics in two-dimensional materials. *ACS Photonics* **8**, 85–101 (2021).

12. A. Vakil, N. Engheta, Transformation optics using graphene. *Science* **332**, 1291–1294 (2011).

13. L. Du, M. R. Molas, Z. Huang, G. Zhang, F. Wang, Z. Sun, Moiré photonics and optoelectronics. *Science* **379**, eadg0014(2023).

14. J. S. Alden, A. W. Tsen, P. Y. Huang, R. Hovden, L. Brown, J. Park, D. A. Muller, P. L. McEuen, Strain solitons and topological defects in bilayer graphene. *Proc. Natl. Acad. Sci. U.S.A.* **110**, 11256–11260 (2013).

15. H. Yoo, R. Engelke, S. Carr, S. Fang, K. Zhang, P. Cazeaux, S. H. Sung, R. Hoyden, A. W. Tsen, T. Taniguchi, K. Watanabe, G.-C. Yi, M. Kim, M. Luskin, E. B. Tadmor, E. Kaxiras, P. Kim, Atomic and electronic reconstruction at the van der Waals interface in twisted bilayer graphene. *Nat. Mater.* **18**, 448–453 (2019).

16. A. Weston, Y. C. Zou, V. Enaldiev, A. Summerfield, N. Clark, V. Zolyomi, A. Graham, C. Yelgel, S. Magorrian, M. W. Zhou, J. Zultak, D. Hopkinson, A. Barinov, T. H. Bointon, A. Kretinin, N. R. Wilsons, P. H. Beton, V. I. Fal'ko, S. J. Haigh, R. Gorbachev, Atomic reconstruction in twisted bilayers of transition metal dichalcogenides. *Nat. Nanotechnol.* **15**, 592–597 (2020).

17. L. J. McGilly, A. Kerelsky, N. R. Finney, K. Shapovalov, E.-M. Shih, A. Ghiotto, Y. H. Zeng, S. L. Moore, W. J. Wu, Y. Bai, K. Watanabe, T. Taniguchi, M. Stengel, L. Zhou, J. Hone, X. Y. Zhu, D. N. Basov, C. Dean, C. E. Dreyer, A. N. Pasupathy, Visualization of moiré superlattices. *Nat. Nanotechnol.* **15**, 580–584 (2020).

18. D. Edelberg, H. Kumar, V. Shenoy, H. Ochoa, A. N. Pasupathy, Tunable strain soliton networks confine electrons in van der Waals materials. *Nat. Phys.* **16**, 1097–1102 (2020).

19. L. L. Jiang, S. Wang, Z. W. Shi, C. H. Jin, M. I. B. Utama, S. H. Zhao, Y.-R. Shen, H. J. Gao, G. Y. Zhang, F. Wang, Manipulation of domain-wall solitons in bi- and trilayer graphene. *Nat. Nanotechnol.* **13**, 204–208 (2018).

20. S. Turkel, J. Swann, Z. Y. Zhu, M. Christos, K. Watanabe, T. Taniguchi, S. Sachdev, M. S. Scheurer, E. Kaxiras, C. R. Dean, A. N. Pasupathy, Orderly disorder in magic-angle twisted trilayer graphene. *Science* **376**, 193–199 (2022).

21. S. S. Sunku, G. X. Ni, B. Y. Jiang, H. Yoo, A. Sternbach, A. S. McLeod, T. Stauber, L. Xiong, T. Taniguchi, K. Watanabe, P. Kim, M. M. Fogler, D. N. Basov, Photonic crystals for nano-light in moiré graphene superlattices. *Science* **362**, 1153–1156 (2018).

22. B.-Y. Jiang, G.-X. Ni, Z. Addison, J. K. Shi, X. Liu, S. Y. F. Zhao, P. Kim, E. J. Mele, D. N. Basov, M. M. Fogler, Plasmon reflections by topological electronic boundaries in bilayer graphene. *Nano Lett.* **17**, 7080–7085 (2017).

23. L. L. Jiang, Z. W. Shi, B. Zeng, S. Wang, J.-H. Kang, T. Joshi, C. Jin, L. Ju, J. Kim, T. Lyu, Y.-R. Shen, M. Crommie, H.-J. Gao, F. Wang, Soliton-dependent plasmon reflection at bilayer graphene domain walls. *Nat. Mater.* **15**, 840–844 (2016).

24. Y. Luo, R. Engelke, M. Mattheakis, M. Tamagnone, S. Carr, K. Watanabe, T. Taniguchi, E. Kaxiras, P. Kim, W. L. Wilson, In situ nanoscale imaging of moire superlattices in twisted van der Waals heterostructures. *Nat. Commun.* **11**, 4209 (2020).

25. L. Brey, T. Stauber, T. Slipchenko, L. Martin-Moreno, Plasmonic Dirac cone in twisted bilayer graphene. *Phys. Rev. Lett.* **125**, 256804 (2020).

26. S. S. Sunku, D. Halbertal, T. Stauber, S. Chen, A. S. McLeod, A. Rikhter, M. E. Berkowitz, C. F. B. Lo, D. E. Gonzalez-Acevedo, J. C. Hone, C. R. Dean, M. M. Fogler, D. N. Basov, Hyperbolic enhancement of photocurrent patterns in minimally twisted bilayer graphene. *Nat. Commun.* **12**, 1641 (2021).

27. N. C. H. Hesp, I. Torre, D. Barcons-Ruiz, H. H. Sheinfux, K. Watanabe, T. Taniguchi, R. K. Kumar, F. H. L. Koppens, Nano-imaging photoresponse in a moire unit cell of minimally twisted bilayer graphene. *Nat. Commun.* **12**, 1640 (2021).

28. K. Chaudhary, M. Tamagnone, X. Yin, C. M. Spägele, S. L. Oscurato, J. Li, C. Persch, R. Li, N. A. Rubin, L. A. Jauregui, K. Watanabe, T. Taniguchi, P. Kim, M. Wuttig, J. H. Edgar, A. Ambrosio, F. Capasso, Polariton nanophotonics using phase-change materials. *Nat. Commun.* **10**, 4487 (2019).

29. Z. Fei, A. S. Rodin, G. O. Andreev, W. Bao, A. S. McLeod, M. Wagner, L. M. Zhang, Z. Zhao, M. Thiemens, G. Dominguez, M. M. Fogler, A. H. Castro Neto, C. N. Lau, F. Keilmann, D. N. Basov, Gate-tuning of graphene plasmons revealed by infrared nano-imaging. *Nature* **487**, 82–85 (2012).

30. J. N. Chen, M. Badioli, P. Alonso-Gonzalez, S. Thongrattanasiri, F. Huth, J. Osmond, M. Spasenovic, A. Centeno, A. Pesquera, P. Godignon, A. Z. Elorza, N. Camara, F. J. G. de Abajo, R. Hillenbrand, F. H. L. Koppens, Optical nano-imaging of gate-tunable graphene plasmons. *Nature* **487**, 77–81 (2012).

31. S. Dai, Q. Ma, M. K. Liu, T. Andersen, Z. Fei, M. D. Goldflam, M. Wagner, K. Watanabe, T. Taniguchi, M. Thiemens, F. Keilmann, G. C. A. M. Janssen, S. E. Zhu, P. Jarillo-Herrero, M. M. Fogler, D. N. Basov, Graphene on hexagonal boron nitride as a tunable hyperbolic metamaterial. *Nat. Nanotechnol.* **10**, 682–686 (2015).

32. S. Dai, Z. Fei, Q. Ma, A. S. Rodin, M. Wagner, A. S. McLeod, M. K. Liu, W. Gannett, W. Regan, K. Watanabe, T. Taniguchi, M. Thiemens, G. Dominguez, A. H. C. Neto, A. Zettl, F. Keilmann, P. Jarillo-Herrero, M. M. Fogler, D. N. Basov, Tunable phonon polaritons in atomically thin van der Waals crystals of boron nitride. *Science* **343**, 1125–1129 (2014).

33. J. D. Caldwell, A. V. Kretinin, Y. G. Chen, V. Giannini, M. M. Fogler, Y. Francescato, C. T. Ellis, J. G. Tischler, C. R. Woods, A. J. Giles, M. Hong, K. Watanabe, T. Taniguchi, S. A. Maier, K. S. Novoselov, Sub-diffractional volume-confined polaritons in the natural hyperbolic material hexagonal boron nitride. *Nat. Commun.* **5**, 5221 (2014).

34. N. P. Kazmierczak, M. Van Winkle, C. Ophus, K. C. Bustillo, S. Carr, H. G. Brown, J. Ciston, T. Taniguchi, K. Watanabe, D. K. Bediako, Strain fields in twisted bilayer graphene. *Nat. Mater.* **20**, 956–963 (2021).

35. F. Zhang, A. H. MacDonald, E. J. Mele, Valley Chern numbers and boundary modes in gapped bilayer graphene. *Proc. Natl. Acad. Sci. U.S.A.* **110**, 10546–10551 (2013).

36. A. Vaezi, Y. Liang, D. H. Ngai, L. Yang, E.-A. Kim, Topological edge states at a tilt boundary in gated multilayer graphene. *Phys. Rev. X* **3**, 021018 (2013).

37. P. Cheben, R. Halir, J. H. Schmid, H. A. Atwater, D. R. Smith, Subwavelength integrated photonics. *Nature* **560**, 565–572 (2018).

38. Q. X. Zhang, T. Senaha, R. L. Zhang, C. Wu, L. Y. Lyu, L. W. Cao, J. Tresback, A. Dai, K. Watanabe, T. Taniguchi, M. T. Allen, Dynamic twisting and imaging of moiré crystals. arXiv:2307.06997 [cond-mat.mes-hall] (2023).

39. C. Hu, T. Wu, X. Huang, Y. Dong, J. Chen, Z. Zhang, B. Lyu, S. Ma, K. Watanabe, T. Taniguchi, G. Xie, X. Li, Q. Liang, Z. Shi, In-situ twistable bilayer graphene. *Sci. Rep.* **12**, 204 (2022).

40. H. Li, S. Li, M. H. Naik, J. Xie, X. Li, J. Wang, E. Regan, D. Wang, W. Zhao, S. Zhao, S. Kahn, K. Yumigeta, M. Blei, T. Taniguchi, K. Watanabe, S. Tongay, A. Zettl, S. G. Louie, F. Wang, M. F. Crommie, Imaging moiré flat bands in three-dimensional reconstructed WSe_2/WS_2 superlattices. *Nat. Mater.* **20**, 945–950 (2021).

41. Z. Zhang, J. Xie, W. Zhao, R. Qi, C. Sanborn, S. Wang, S. Kahn, K. Watanabe, T. Taniguchi, A. Zettl, M. Crommie, F. Wang, Engineering correlated insulators in bilayer graphene with a remote Coulomb superlattice. *Nat. Mater.* **23**, 189–195 (2024).

42. L. Wang, I. Meric, P. Y. Huang, Q. Gao, Y. Gao, H. Tran, T. Taniguchi, K. Watanabe, L. M. Campos, D. A. Muller, J. Guo, P. Kim, J. Hone, K. L. Shepard, C. R. Dean, One-dimensional electrical contact to a two-dimensional material. *Science* **342**, 614–617 (2013).

43. K. Kim, M. Yankowitz, B. Fallahazad, S. Kang, H. C. P. Movva, S. Huang, S. Larentis, C. M. Corbet, T. Taniguchi, K. Watanabe, S. K. Banerjee, B. J. LeRoy, E. Tutuc, van der Waals heterostructures with high accuracy rotational alignment. *Nano Lett.* **16**, 1989–1995 (2016).

44. M. Koshino, Electronic transmission through AB-BA domain boundary in bilayer graphene. *Phys. Rev. B* **88**, 115409 (2013).

45. P. San-Jose, R. V. Gorbachev, A. K. Geim, K. S. Novoselov, F. Guinea, Stacking boundaries and transport in bilayer graphene. *Nano Lett.* **14**, 2052–2057 (2014).

46. Z. Fei, E. G. Iwinski, G. X. Ni, L. M. Zhang, W. Bao, A. S. Rodin, Y. Lee, M. Wagner, M. K. Liu, S. Dai, M. D. Goldflam, M. Thiemens, F. Keilmann, C. N. Lau, A. H. Castro-Neto, M. M. Fogler, D. N. Basov, Tunneling plasmonics in bilayer graphene. *Nano Lett.* **15**, 4973–4978 (2015).

47. X. Chen, Z. Yao, S. G. Stanciu, D. N. Basov, R. Hillenbrand, M. Liu, Rapid simulations of hyperspectral near-field images of three-dimensional heterogeneous surfaces. *Opt. Express* **29**, 39648–39668 (2021).

48. X. Chen, Z. Yao, S. G. Stanciu, D. N. Basov, R. Hillenbrand, M. Liu, Rapid simulations of hyperspectral near-field images of three-dimensional heterogeneous surfaces—Part II. *Opt. Express* **30**, 11228–11242 (2022).

49. V. Semenenko, M. K. Liu, V. Perebeinos, Simulation of scanning near-field optical microscopy spectra of 1D plasmonic graphene junctions. *Opt. Express* **30**, 9000–9007 (2022).

50. E. D. Palik, *Handbook of Optical Constants of Solids* (Academic Press, 1985), pp. 719–752.

51. J. Kischkat, S. Peters, B. Gruska, M. Semtsiv, M. Chashnikova, M. Klinkmuller, O. Fedosenko, S. Machulik, A. Aleksandrova, G. Monastyrskyi, Y. Flores, W. T. Masselink, Mid-infrared optical properties of thin films of aluminum oxide, titanium dioxide, silicon dioxide, aluminum nitride, and silicon nitride. *Appl. Opt.* **51**, 6789–6798 (2012).

52. A. Woessner, M. B. Lundeberg, Y. Gao, A. Principi, P. Alonso-Gonzalez, M. Carrega, K. Watanabe, T. Taniguchi, G. Vignale, M. Polini, J. Hone, R. Hillenbrand, F. H. Koppens, Highly confined low-loss plasmons in graphene-boron nitride heterostructures. *Nat. Mater.* **14**, 421–425 (2015).
